# Clinical parameter-based prediction model for neurosyphilis risk stratification

**DOI:** 10.1017/S0950268824000074

**Published:** 2024-01-15

**Authors:** Yilan Yang, Xin Gu, Lin Zhu, Yuanyuan Cheng, Haikong Lu, Zhifang Guan, Mei Shi, Liyan Ni, Ruirui Peng, Wei Zhao, Juan Wu, Tengfei Qi, Fuquan Long, Zhe Chai, Weiming Gong, Meiping Ye, Pingyu Zhou

**Affiliations:** Institute of Sexually Transmitted Disease, Shanghai Skin Disease Hospital, School of Medicine, Tongji University, Shanghai, China

**Keywords:** lumbar puncture, neurological, neurosyphilis, psychiatric symptoms

## Abstract

Accurately predicting neurosyphilis prior to a lumbar puncture (LP) is critical for the prompt management of neurosyphilis. However, a valid and reliable model for this purpose is still lacking. This study aimed to develop a nomogram for the accurate identification of neurosyphilis in patients with syphilis. The training cohort included 9,504 syphilis patients who underwent initial neurosyphilis evaluation between 2009 and 2020, while the validation cohort comprised 526 patients whose data were prospectively collected from January 2021 to September 2021. Neurosyphilis was observed in 35.8% (3,400/9,504) of the training cohort and 37.6% (198/526) of the validation cohort. The nomogram incorporated factors such as age, male gender, neurological and psychiatric symptoms, serum RPR, a mucous plaque of the larynx and nose, a history of other STD infections, and co-diabetes. The model exhibited good performance with concordance indexes of 0.84 (95% CI, 0.83–0.85) and 0.82 (95% CI, 0.78–0.86) in the training and validation cohorts, respectively, along with well-fitted calibration curves. This study developed a precise nomogram to predict neurosyphilis risk in syphilis patients, with potential implications for early detection prior to an LP.

## Introduction

Neurosyphilis is a significant global health problem [1], with an annual incidence ranging from 0.47 to 2.1 cases per 100,000 adults in various studies [2, 3]. Patients who do not receive timely treatment can experience irreversible neurological damage and even life-threatening consequences [4, 5]. Therefore, an early diagnosis of neurosyphilis offers significant benefits in preventing irreversible neurological damage. However, a confirmed diagnosis of neurosyphilis primarily relies on abnormal cerebrospinal fluid (CSF) findings [6], and a routine lumbar puncture (LP) is not recommended in many cases due to its unpleasant nature [7]. Consequently, the diagnosis has limited impact on decision-making prior to an LP. Accurately estimating the risk of neurosyphilis prior to an LP may assist clinicians in providing early treatment to patients based on risk–benefit assessment.

Numerous studies have been conducted to assess the risk of neurosyphilis. Several authors have suggested that age and gender play a role in neurosyphilis [2, 8–11]. Our previous research has demonstrated that being male and aged over 45 years are correlated risk factors for neurosyphilis. Furthermore, the risk of neurosyphilis tends to increase with higher serum rapid plasma reagin (RPR) titres [10]. Some studies have proposed the use of serum biomarkers to estimate the risk of neurosyphilis [12, 13]. Additionally, some authors have reported that a history of diabetes could be related to neurosyphilis [14]. Although certain factors have been associated with neurosyphilis, their clinical applicability still needs to be determined. It is now imperative to develop a more accurate scoring system that incorporates the risk factors of neurosyphilis to predict its occurrence.

Due to the lack of a practical and specific predictive method, the development of a predictive model that factors in variables associated with neurosyphilis prior to an LP becomes necessary. A nomogram is a graphical statistical instrument that provides a simple and visual method for estimating or determining values based on known variables. It can precisely calculate the risk for every patient. To our knowledge, we have established a nomogram based on the largest sample for neurosyphilis risk estimation prior to an LP.

## Materials and methods

### Study population and data collection

We conducted a retrospective cohort study involving 9,504 consecutive patients with syphilis who underwent initial neurosyphilis evaluation at the Department of Sexually Transmitted Disease in the Shanghai Skin Disease Hospital, Tongji University School of Medicine, Shanghai, China, between 2009 and 2020. The data for the validation cohort were prospectively collected from January 2021 to September 2021. Neurosyphilis or non-neurosyphilis diagnoses were based on the clinical assessment [15–17] and applied to HIV-negative syphilis patients. Prior to an LP, all enrolled patients had not received any anti-neurosyphilitic treatment. Both central and peripheral nervous system functions were routinely examined. The exclusion criteria comprised pregnant or lactating women, patients with a failed LP, and those with CSF containing blood, bacterial, or fungal infection. Additionally, cases of ocular, aural, cardiovascular, and osteal syphilis were excluded, along with those with incomplete clinical data. The collaborative differential diagnosis conducted by a multidisciplinary team including a neurologist, psychiatrist, dermatologist, and physician, along with the examination using CT or MRI, helped eliminate individuals with neurological or psychiatric symptoms attributed to other diseases. Furthermore, individuals with a family history of neurological and psychiatric symptoms were also excluded.

.

### Diagnosis of neurosyphilis and clinical variables

Peripheral venous blood was routinely collected from all patients, and an LP was performed on syphilis patients in accordance with US, European, and Chinese national guidelines [15–17]. The details of the LP criteria were as follows: 1) syphilis patients with neurological or psychiatric symptoms; 2) syphilis patients without neurological or psychiatric symptoms, if a) nontreponemal test titres of patients with treatment failure do not decrease by at least fourfold within 12 months after therapy or their nontreponemal test titres shift from negative to positive or increased by more than fourfold compared to the latest treatment levels and b) the patient requests neurosyphilis evaluation due to persistent positive results on the nontreponemal test (i.e. the test remains positive despite a sustained decline) after at least a year of anti-syphilitic treatment. Under the collaborative diagnostic assessment of multidisciplinary medical professionals, an LP is considered beneficial to uncover the progress of the disease.

The laboratory diagnostic criteria for neurosyphilis included 1) reactive treponema and non-treponema tests in peripheral blood (screening for syphilis using an RPR test and confirmed by a *T. pallidum* particle agglutination test), along with a reactive cerebrospinal fluid–venereal disease research laboratory (CSF-VDRL) test, and/or 2) a CSF protein concentration higher than 50 mg/dL and/or a white blood cell count exceeding 10 white blood cells/μL in the absence of other known causes of these abnormalities, as previously reported [10, 15–17].

Neurological symptoms comprised fever, dizziness, headache, nausea and vomiting, epileptic seizures, bowel and urine dysfunction, gait ataxia, paralysis, candy sign, a sensation of walking on cotton, sensory disorders in the limbs, limb tremors, lightning-like pain, limb weakness, limb anaesthesia, girdle sensation, hypotonia, lower limb oedema, a feeling of walking on cobblestones, and Charcot joints. Psychiatric symptoms included disorders of consciousness, cognitive disorders, thought disorders, affective disorders, volitional behaviour disorders, memory disorders, intelligence disorders, insight, allolalia, dysarthria, and dyssomnia.

Other sexually transmitted diseases (STDs) included gonorrhoea, chlamydia, mycoplasma, genital herpes, and condyloma acuminata. Peripheral blood and CSF were routinely collected from all patients, and STD tests carried out. All laboratory tests were performed at the central laboratory of the Shanghai Skin Disease Hospital.

### Statistical analysis

Continuous variables were presented as median (interquartile range), while categorical variables were presented as *n* (%). Baseline data comparisons were performed between patients with and without neurosyphilis, as well as between patients in the training and validation cohorts. Mann–Whitney U-tests were used for comparing continuous variables with nonparametric data, and chi-squared tests were used for comparing categorical variables. Univariate and multivariate logistic regression analyses were conducted to identify risk factors associated with neurosyphilis.

Multivariate logistic regression analysis was performed to establish the nomogram. The nomogram was constructed using data from the training cohort and externally validated using the data from the validation cohort. Each coefficient in the multivariate logistic regression was converted proportionally to a 0- to 100-point scale on the nomogram. The variable with the highest β coefficient was assigned 100 points. The scores of each independent variable were summed to obtain the total score, which was then converted to corresponding predicted probabilities. The accuracy of the nomogram was evaluated by the receiver operating characteristic (ROC) curve. The calibration of the nomogram was assessed using the calibration curve and Hosmer–Lemeshow test. Decision curve analysis (DCA) was employed to evaluate the validity of the nomogram.

For the clinical use of this model, the total scores of every patient were calculated based on the neurosyphilis nomogram. The ROC curve was used to calculate the cut-off values that were determined by the Youden index (i.e. sensitivity+specificity−1). The accuracy of the cut-off value was assessed by the specificity, sensitivity, predictive values, and likelihood ratios.

Statistical analyses were conducted using R software (Comprehensive R Archive Network Project 4.1.1) and SPSS 26.0 statistical software (IBM, Armonk, NY). A two-tailed value of *P* < 0.05 was considered statistically significant.

## Results

### Baseline characteristics

A total of 9,504 syphilis patients (age: 48 (33–59) years; 53.1% male) were recruited to the training cohort, while 526 patients (age: 51 (32–62) years; 57.2% male) were included in the validation cohort. The baseline characteristics of the patients with and without neurosyphilis in the training cohort are listed in Table 1. It was observed that the neurosyphilis group consisted of older individuals and a higher proportion of males. Additionally, the neurosyphilis group had a significantly higher serum RPR titre than the non-neurosyphilis group.

**Table 1.** Baseline demographic and clinical characteristics of all patients

	Neurosyphilis	Non-neurosyphilis	*P*-value
Patients, *n*	3,400	6,104	
Age, years	56 (47–62)	40 (30–55)	<0.001
Gender, *n* (%)			<0.001
Male	2,426 (71.4)	2,621 (42.9)	
Female	974 (28.6)	3,483 (57.1)	
Sexual orientation, *n* (%)			<0.001
MSM	41 (1.2)	196 (3.2)	
Heterosexual	3,359 (98.8)	5,908 (96.8)	
Clinical symptoms, *n* (%)			
Neurological^a^	968 (28.5)	210 (3.2)	<0.001
Psychiatric^b^	1,028 (30.2)	27 (0.4)	<0.001
Serum RPR titre, *n* (%)			<0.001
≤1: 8	644 (18.9)	2,664 (43.6)	
1:16	625 (18.4)	999 (16.4)	
1:32	799 (23.5)	1,031 (16.9)	
1:64	774 (22.8)	895 (14.7)	
≥1:128	558 (16.4)	515 (8.4)	
Skin lesion, *n* (%)			
Chancre	124 (3.6)	523 (8.6)	<0.001
Palmoplantar rash	123 (3.6)	504 (8.3)	<0.001
Trunk or limb rash	219 (6.4)	1,011 (16.6)	<0.001
Condyloma	102 (3.0)	316 (5.2)	0.304
Mucous plaque of the larynx and nose	26 (0.8)	21 (0.3)	0.003
Mucous plaque of oral	70 (2.1)	151 (2.5)	0.230
History of other STD infections, *n* (%)	1,197 (35.2)	1,472 (24.1)	<0.001
Comorbid conditions, *n* (%)			
Diabetes	555 (16.3)	452 (7.4)	<0.001
Autoimmune disease	320 (9.4)	942 (15.4)	<0.001
Hepatitis	280 (8.2)	532 (8.7)	0.405
Cancer	348 (10.2)	1,003 (16.4)	<0.001

Data are presented as *n* (%) or median (interquartile range). MSM, men who have sex with men; RPR, rapid plasma reagin; STD, sexually transmitted disease.

a Neurological symptoms only include fever, dizziness, headache, nausea and vomiting, epileptic seizure, bowel and urine dysfunction, gait ataxia, paralysis, candy sign, feeling of walking on cotton, sensory disorder in the limbs, limb tremor, lightning-like pain, limb weakness and anaesthesia, girdle sensation, hypotonia, lower limb oedema, feeling of walking on cobblestone, and Charcot joints.

b Psychiatric symptoms only include disorder of consciousness, cognitive disorder, thought disorder, affective disorder, volitional behaviour disorder, memory disorder, intelligence disorder, insight, allolalia, dysarthria, and dyssomnia.

Other STDs include gonorrhoea, chlamydia, mycoplasma, genital herpes, and condyloma acuminata.

Comparisons between the training cohort and the validation cohort revealed significant differences in age, sexual orientation, psychiatric symptoms, serum RPR titres, the presence of trunk or limb rashes, condyloma, mucous plaque of oral, a history of autoimmune disease, hepatitis, and cancer (Table 2).

**Table 2.** Baseline demographic and clinical characteristics of the training and validation cohorts

	Training cohort (*n* = 9,504)	Validation cohort (*n* = 526)	*P*-value
Neurosyphilis, *n* (%)	3,400 (35.8)	198 (37.6)	0.345
Age, years	48 (33–59)	51 (32–62)	0.03
Gender, *n* (%)			0.065
Male	5,047 (53.1)	301 (57.2)	
Female	4,457 (46.9)	225 (42.8)	
Sexual orientation, *n* (%)			<0.001
MSM	237 (2.5)	45 (9.1)	
Heterosexual	9,267 (97.5)	481 (91.4)	
Clinical symptoms, *n* (%)			
Neurological^a^	1,178 (12.4)	70 (13.3)	0.537
Psychiatric^b^	1,055 (11.1)	76 (14.4)	0.018
Serum RPR titre, *n* (%)			0.005
≤1: 8	3,308 (34.8)	197 (37.5)	
1:16	1,624 (17.0)	97 (18.4)	
1:32	1830 (19.2)	114 (21.7)	
1:64	1,669 (17.5)	74 (14.1)	
≥1:128	1,073 (11.2)	44 (8.4)	
Skin lesion, *n* (%)			
Chancre	647 (6.8)	27 (5.1)	0.135
Palmoplantar rash	627 (6.6)	40 (7.6)	0.367
Trunk or limb rash	1,230 (12.9)	52 (9.9)	0.041
Condyloma	418 (4.4)	13 (2.5)	0.049
Mucous plaque of the larynx and nose	47 (0.5)	1 (0.2)	0.325
Mucous plaque of oral	221 (2.3)	6 (1.1)	0.075
History of other STD infections, *n* (%)	2,669 (28.1)	156 (29.7)	0.434
Comorbid conditions, *n* (%)			
Diabetes	1,007 (10.6)	45 (8.6)	0.137
Autoimmune disease	1,262 (13.3)	2 (0.4)	<0.001
Hepatitis	812 (8.5)	19 (3.6)	<0.001
Cancer	1,351 (14.2)	11 (2.1)	<0.001

Data are presented as *n* (%) or median (interquartile range). MSM, men who have sex with men; RPR, rapid plasma reagin; STD, sexually transmitted disease.

a Neurological symptoms only include fever, dizziness, headache, nausea and vomiting, epileptic seizure, bowel and urine dysfunction, gait ataxia, paralysis, candy sign, feeling of walking on cotton, sensory disorder in the limbs, limb tremor, lightning-like pain, limb weakness and anaesthesia, girdle sensation, hypotonia, lower limb oedema, feeling of walking on cobblestone, and Charcot joints.

b Psychiatric symptoms only include disorder of consciousness, cognitive disorder, thought disorder, affective disorder, volitional behaviour disorder, memory disorder, intelligence disorder, insight, allolalia, dysarthria, and dyssomnia.

Other STDs include gonorrhoea, chlamydia, mycoplasma, genital herpes, and condyloma acuminata.

### Development and validation of a neurosyphilis-predicting nomogram

Univariate and multivariate analyses of clinical characteristics in the training cohort revealed that age, serum RPR titre, male gender, the presence of neurological or psychiatric symptoms, mucous plaque of the larynx and nose, a history of other STD infections, and co-diabetes were significant predictors (Tables 3 and 4).

**Table 3.** Univariate logistic regression analysis of correlated factors for neurosyphilis patients

Variable	OR (95% CI)	*P*-value
Gender, male vs. female	2.92 (2.67–3.19)	<0.001
Age, years (vs ≤ 30)		
31–40	1.99 (1.67–2.38)	<0.001
41–50	5.14 (4.34–6.09)	<0.001
51–60	9.76 (8.32–11.45)	<0.001
>60	9.73 (8.26–11.44)	<0.001
MSM	0.44 (0.37–0.52)	<0.001
Neurological symptoms^a^	11.46 (9.82–13.38)	<0.001
Psychiatric symptoms^b^	97.02 (66.47–141.62)	<0.001
Serum RPR titre (vs ≤ 1:8)		
1:16	2.48 (2.18–2.84)	<0.001
1:32	3.05 (2.69–3.45)	<0.001
1:64	3.25 (2.87–3.69)	<0.001
≥1:128	3.68 (3.22–4.23)	<0.001
Skin lesion		
Chancre	0.41 (0.34–0.50)	<0.001
Palmoplantar rash	0.41 (0.34–0.49)	<0.001
Trunk or limb rash	0.34 (0.30–0.39)	<0.001
Condyloma	0.65 (0.53–0.80)	<0.001
Mucous plaque of the larynx and nose	2.31 (1.32–4.07)	0.004
Mucous plaque of oral	0.85 (0.65–1.11)	0.230
History of other STD infections, *n* (%)	1.66 (1.52–1.81)	<0.001
Comorbid conditions, *n* (%)		
Diabetes	2.83 (2.46–3.26)	<0.001
Autoimmune disease	0.59 (0.52–0.68)	<0.001
Hepatitis	0.94 (0.81–1.09)	0.405
Cancer	0.61 (0.54–0.70)	<0.001

CI, confidence interval; MSM, men who have sex with men; OR, odds ratio; RPR, rapid plasma reagin; STD, sexually transmitted disease.

a Neurological symptoms only include fever, dizziness, headache, nausea and vomiting, epileptic seizure, bowel and urine dysfunction, gait ataxia, paralysis, candy sign, feeling of walking on cotton, sensory disorder in the limbs, limb tremor, lightning-like pain, limb weakness and anaesthesia, girdle sensation, hypotonia, lower limb oedema, feeling of walking on cobblestone, and Charcot joints.

b Psychiatric symptoms only include disorder of consciousness, cognitive disorder, thought disorder, affective disorder, volitional behaviour disorder, memory disorder, intelligence disorder, insight, allolalia, dysarthria, and dyssomnia.

Other STDs include gonorrhoea, chlamydia, mycoplasma, genital herpes, and condyloma acuminata.

**Table 4.** Multivariate logistic regression analysis of correlated factors for neurosyphilis patients

Variable	β	OR (95% CI)	*P*-value
Gender, male vs. female	0.55	1.73 (1.56–1.93)	<0.001
Age, years (vs ≤ 30)			
31–40	0.46	1.58 (1.31–1.92)	<0.001
41–50	1.24	3.44 (2.86–4.14)	<0.001
51–60	1.68	5.37 (4.49–6.37)	<0.001
>60	1.72	5.57 (4.65–6.83)	<0.001
Neurological symptoms^a^	1.61	4.99 (4.14–6.03)	<0.001
Psychiatric symptoms^b^	3.84	46.47 (31.43–68.72)	<0.001
Serum RPR titre (vs ≤ 1:8)			
1:16	1.07	2.93 (2.49–3.45)	<0.001
1:32	1.27	3.57 (3.06–4.17)	<0.001
1:64	1.38	3.98 (3.40–4.66)	<0.001
≥1:128	1.56	4.75 (4.02–5.61)	<0.001
Mucous plaque of the larynx and nose	0.66	1.94 (1.06–3.53)	0.001
History of other STD infections	0.31	1.37 (1.22–1.53)	<0.001
Diabetes	0.89	2.45 (2.15–1.87)	<0.001

CI, confidence interval; OR, odds ratio; RPR, rapid plasma reagin; STD, sexually transmitted disease.

a Neurological symptoms only include fever, dizziness, headache, nausea and vomiting, epileptic seizure, bowel and urine dysfunction, gait ataxia, paralysis, candy sign, feeling of walking on cotton, sensory disorder in the limbs, limb tremor, lightning-like pain, limb weakness and anaesthesia, girdle sensation, hypotonia, lower limb oedema, feeling of walking on cobblestone, and Charcot joints.

b Psychiatric symptoms only include disorder of consciousness, cognitive disorder, thought disorder, affective disorder, volitional behaviour disorder, memory disorder, intelligence disorder, insight, allolalia, dysarthria, and dyssomnia.

Other STDs include gonorrhoea, chlamydia, mycoplasma, genital herpes, and condyloma acuminata.

**Figure 1.**
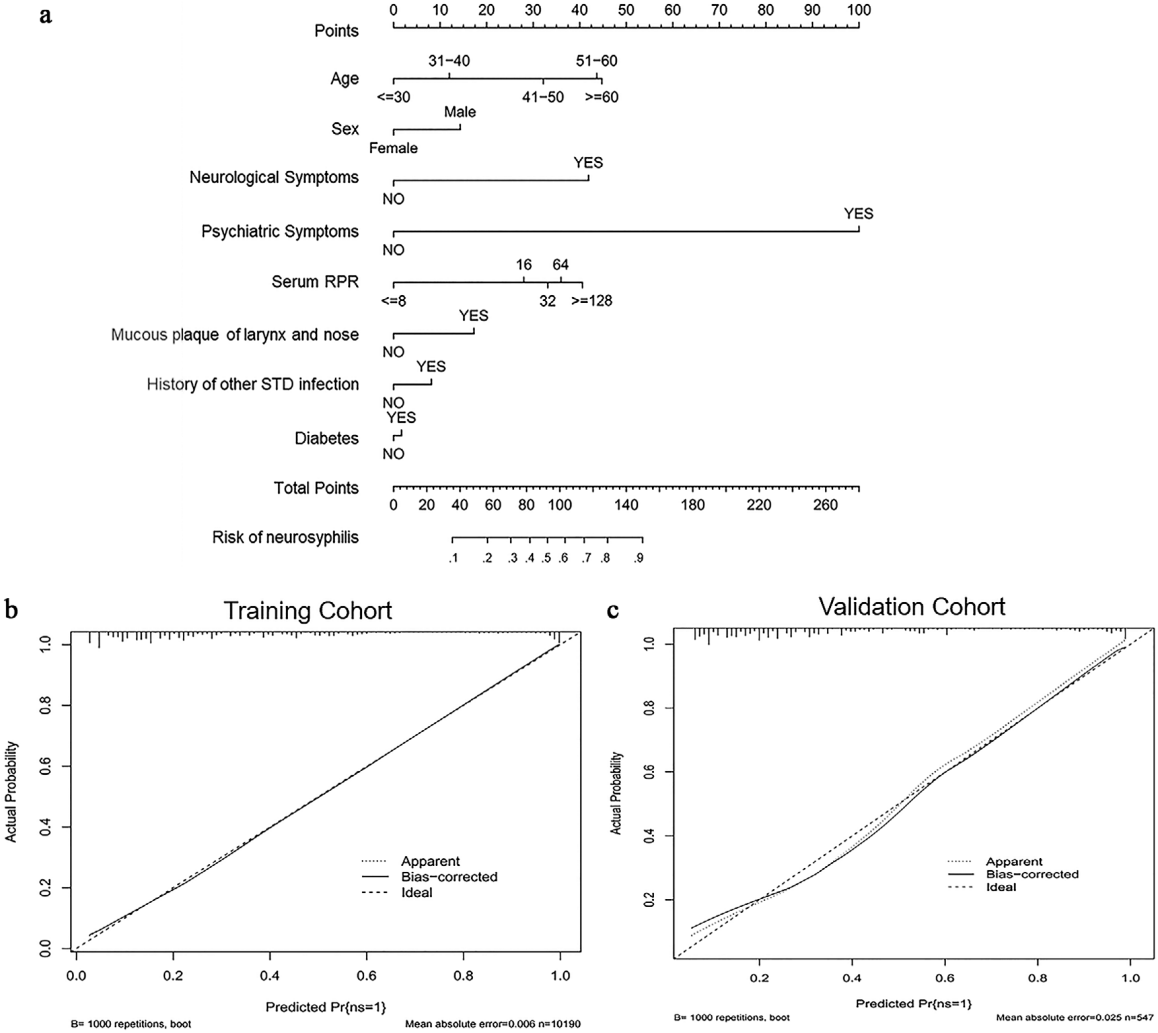
Development and performance of the nomogram to predict the risk of neurosyphilis. (a) Nomogram based on clinical factors; (b,c) calibration plot of the nomogram in the training (b) and validation cohorts (c). The 45° line in the plot indicates a perfect calibration that the predictive capability of the model perfectly matches the actual risk of neurosyphilis. The dotted line represents the performance of the nomogram, while the solid line corrects for any bias in the nomogram.

**Figure 2.**
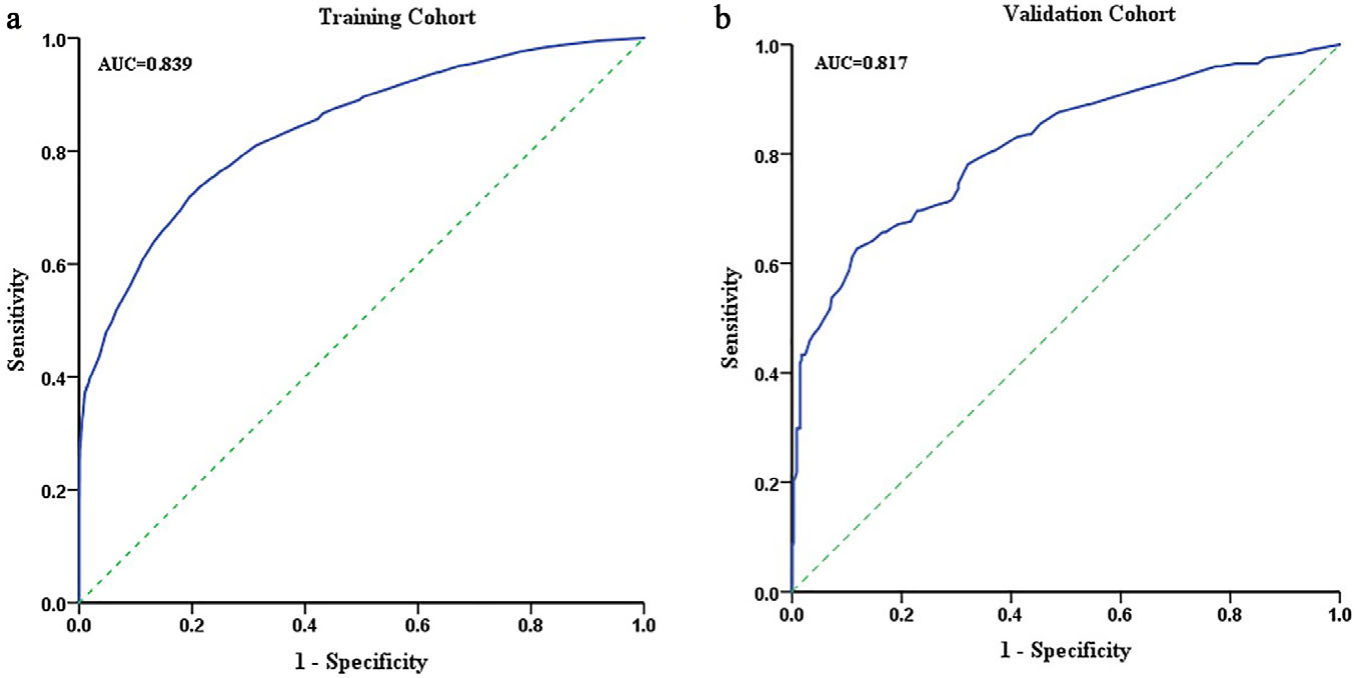
ROC curve of the nomogram in the training (a) and validation cohorts (b).

Based on these independent risk factors, a neurosyphilis risk estimation nomogram was constructed (Figure 1). The model exhibited concordance indexes of 0.84 (95% CI, 0.83–0.85) and 0.82 (95% CI, 0.78–0.86) in the training and validation cohorts, respectively (Table 5; Figure 2). Figure 1 illustrates a calibration plot for risk estimation, with mean absolute errors of 0.006 and 0.025 for the training and validation cohorts, respectively. The calibration curve demonstrates a good agreement between the training and validation cohorts (Figure 1). The Hosmer–Lemeshow test was not significant (*P* > 0.05), demonstrating a good fit.

**Table 5.** Accuracy of the prediction score of the nomogram for estimating the risk of neurosyphilis

Variable	Training cohort	Validation cohort
Area under ROC curve	0.84 (0.83–0.85)	0.82 (0.78–0.86)
Cut-off score	75.25	75.25
Sensitivity, %	0.71	0.70
Specificity, %	0.81	0.77
Positive predictive value, %	0.67	0.64
Negative predictive value, %	0.83	0.81
Positive likelihood ratio	3.74	3.04
Negative likelihood ratio	0.36	0.39

ROC, receiver operating characteristic.

The nomogram can also be used to screen asymptomatic neurosyphilis patients among all syphilis patients. To demonstrate this feasibility, we excluded patients with neurological or psychiatric symptoms, resulting in two patient groups: non-neurosyphilis and asymptomatic neurosyphilis (figures in Supplementary material). Therefore, it can be confirmed that the nomogram is also suitable for assessing the risk of neurosyphilis in syphilis patients without neurological or psychiatric symptoms.

### Risk of neurosyphilis based on nomogram scores

**Figure 3.**
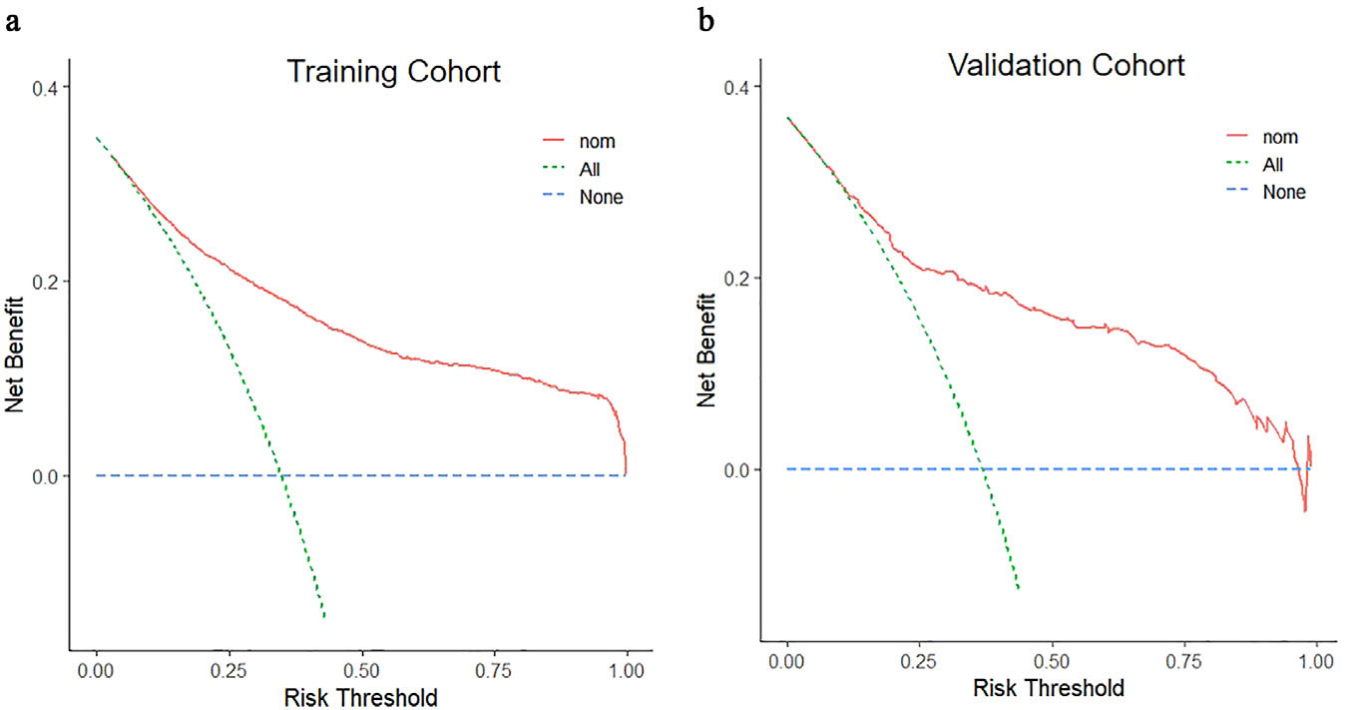
Decision curve analysis of the nomogram in the training (a) and validation cohorts (b). The x-axis denotes the threshold probability, while the y-axis denotes the net benefit. The green line represents that all syphilis patients developed neurosyphilis, the blue line represents that no syphilis patients developed neurosyphilis, and the red line represents the nomogram to predict neurosyphilis in patients with syphilis.

The decision curve was used to assess the clinical benefit of the neurosyphilis nomogram, performing an LP (all-LP) and not performing (none-LP) (Figure 3).

These patients would benefit more from the neurosyphilis nomogram than either of the all-LP or none-LP strategies.

Furthermore, the optimal cut-off value of the nomogram scores was determined to be 75.25. The specificity, sensitivity, negative predictive value, and positive predictive value when used in differentiating neurosyphilis were 81%, 71%, 83%, and 67% in the training cohort and 77%, 70%, 81%, and 64% in the validation cohort, respectively (Table 5).

## Discussion

Early diagnosis and timely treatment of neurosyphilis are crucial to preventing irreversible damage to the central nervous system (CNS) [5]. The clinical spectrum of neurosyphilis in China differs significantly from that in Western countries, where neurological or psychiatric symptoms are the primary indications for performing an LP in syphilis patients [18]. It is important to note that benzathine penicillin cannot penetrate the blood–brain barrier (BBB) [19] and symptomatic neurosyphilis can occur even in patients who have received appropriate treatment and have shown a good response [20]. In China, the guidelines recommend performing an LP for syphilis patients beyond those presenting with neurological or psychiatric symptoms. This approach highlights the need to consider neurosyphilis in a broader range of cases to ensure early detection and appropriate management.

The presence of neurological and psychiatric symptoms in patients with syphilis indicates damage to the CNS and is strongly associated with neurosyphilis [9, 21]. The observations of our study, which involved a large sample size of 9,504 syphilis patients, reinforce the importance of considering neurosyphilis as a potential cause in patients presenting with neuropsychiatric symptoms without other known causes. This highlights the need for clinicians to have a high index of suspicion for neurosyphilis in such cases to ensure timely diagnosis and appropriate management.

An interesting finding in this study is that mucous lesions can predict the risk of neurosyphilis. Our recently published study found that *T. pallidum* can enter the bloodstream first after infection and then spread to the mouth and be preserved in saliva. The detection of *T. pallidum* DNA in saliva at any stage of syphilis and a higher detection rate compared to plasma suggest the potential involvement of the oral mucosa as a reservoir for *T. pallidum* [22]. This significant value of mucous lesions occurring in the larynx and the nose in predicting the risk of neurosyphilis may be explained by this phenomenon.

The novel finding that a history of infection with other STDs can predict neurosyphilis has not been reported previously. The potential increased risk of neurosyphilis associated with STDs can be speculated based on several factors. Firstly, STDs may disrupt the normal epithelial barrier of genital organs, leading to increased shedding of *T. pallidum* in the genital tract, similar to how STDs enhance susceptibility to HIV [23]. Secondly, STDs may alter the cytokine milieu, potentially increasing the plasma concentration of *T. pallidum*, which could contribute to a more rapid progression of neurosyphilis [24]. Moreover, the impact of pathogens on human immunity can lead to the destruction of immune cells and impair the defence function of the immune system. Pathogens may have specific pathological mechanisms that allow them to evade the autophagy-killing effect of the host cell [25–28].

Moreover, a wealth of research has consistently demonstrated that male patients who are older and have higher serum RPR levels during certain stages of syphilis are at a greater risk of developing neurosyphilis [10, 11, 29]. These findings align with the results of our study. Additionally, recent smaller studies have indicated a positive association between diabetes and neurosyphilis [30, 31]. Notably, our study confirms these findings and offers a plausible explanation: diabetes may contribute to dysfunction of the BBB [32].

The use of the nomogram in evaluating the risk of a patient harbouring neurosyphilis to direct CSF examination and clinical anti-neurosyphilitic treatment is a new concept. Based on these prior to LP predictions, the nomogram could serve as a tool to provide LP recommendations for patients with different risks of neurosyphilis. A clear understanding of the model and its application will assist clinicians in clinical practice. An illustrative example of the model’s usage is observed in a hypothetical 40-year-old male patient admitted with a serum RPR level of 1:8, neurological symptoms, mucous plaque of the larynx and nose, a history of other STD infections, and co-diabetes. In this patient, the scores for age, gender, serum RPR level of 1:8, neurological symptoms, mucous plaque of the larynx and nose, a history of other STD infections, and co-diabetes were 32, 40, 0, 116, 46, 22, and 4, respectively. The total score for this patient was 260, indicating a high risk of neurosyphilis.

By identifying high-risk patients with neurosyphilis through this model, doctors can achieve a theoretical foundation for more targeted screening of suitable candidates for an LP, facilitating the timely diagnosis of neurosyphilis. For non-neurosyphilis high-risk patients, an active follow-up is deemed essential to minimize subjective judgment and broaden the scope of LP. Furthermore, it can aid in determining the appropriateness of LP for further confirmation in a hospital setting. By predicting neurosyphilis before LP, we can not only reduce the potential for severe outcomes in neurosyphilis patients but also prevent unnecessary LP procedures and conserve valuable hospital resources for patients who are unlikely to have neurosyphilis.

It is important to acknowledge the limitations of this study. Firstly, due to the prospective collection of patients in the validation group, ensuring complete consistency across numerous variables is challenging. The analysis was based on a single institution, which may introduce selection bias and limit the generalizability of the findings. Secondly, the study did not conduct a specific analysis of clinical symptoms, which are essential for clinical decision-making. Future research should focus on evaluating the predictive value of specific symptoms for neurosyphilis as clinicians play a vital role in interpreting and utilizing clinical information. Thirdly, the sensitivity of this model was 70% to estimate the risk of neurosyphilis before LP in the validation cohort, which has great clinical value. However, it still had a 30% omission diagnostic rate. In future, to enhance the predictive accuracy, we plan to conduct prospective trials incorporating more indicators and patients. Lastly, it is worth noting that the enrolled participants in both cohorts were Chinese patients with syphilis. To establish the universality of the model, it is necessary to include a more diverse population in order to validate its performance in different settings and populations.

## Conclusion

This study presents a nomogram based on clinical characteristics for predicting occult neurosyphilis. This nomogram holds great potential for clinical practice, providing valuable insights into individualized follow-up strategies and treatment options for patients with syphilis. By incorporating key risk factors, the nomogram offers a practical tool to identify individuals at higher risk of developing neurosyphilis, facilitating timely interventions and optimizing patient care.

## Supporting information

Yang et al. supplementary material Yang et al. supplementary material

## Data Availability

The datasets generated during and/or analysed during the current study are available from the corresponding author on reasonable request.

## Supplementary material

For supplementary material accompanying this paper visit http://doi.org/10.1017/S0950268824000074. click here to view supplementary material
