# Supplementary figures and images for: Clinical parameter-based prediction model for neurosyphilis risk stratification
Source: Epidemiol Infect. 2024 Jan 15;152:e21. doi: 10.1017/S0950268824000074 (PMC10894895; doi:10.1017/S0950268824000074)

**Supporting data**

Figure S1. ROC curve of the nomogram in asymptomatic neurosyphilis cohort.


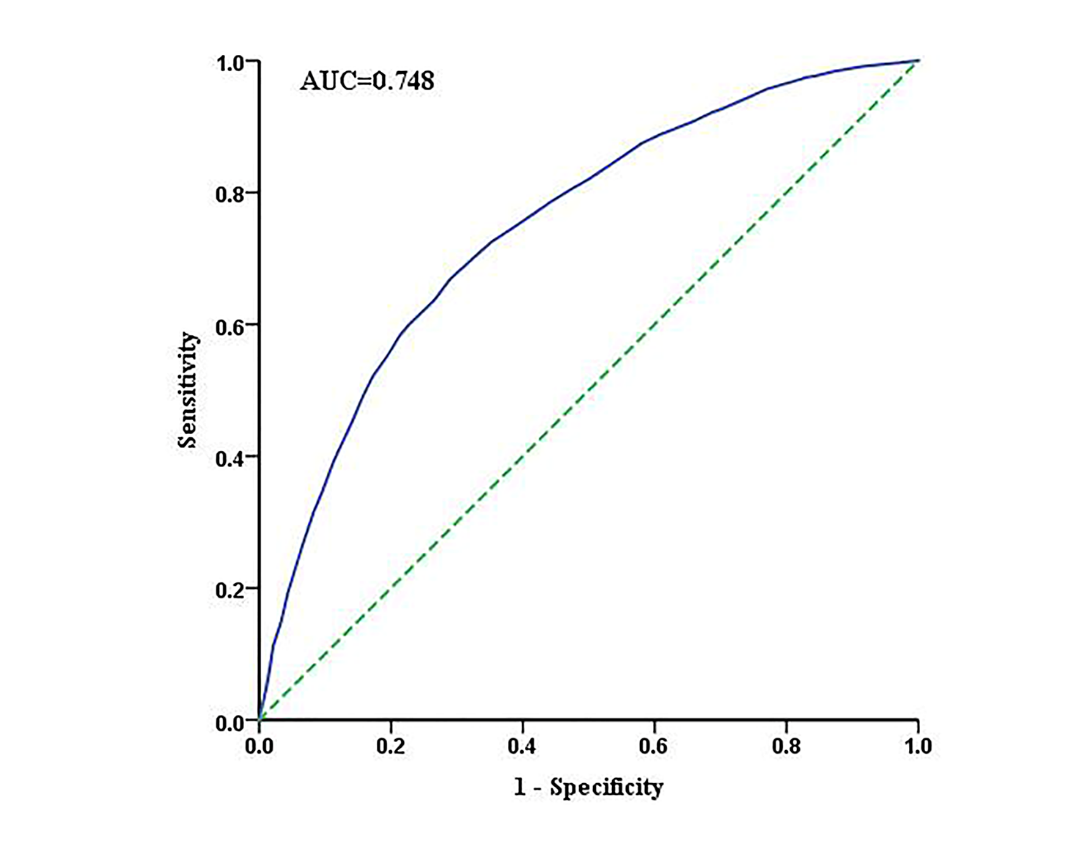

Supplement: Yang et al. supplementary material [file S0950268824000074sup001.docx]
